# The impact of obesity and hypoxia on left ventricular function and glycolytic metabolism

**DOI:** 10.14814/phy2.12001

**Published:** 2014-03-26

**Authors:** Rosa H. Rodriguez, Janelle L. Bickta, Patrick Murawski, Christopher P. O'Donnell

**Affiliations:** 1 Division of Pulmonary, Allergy and Critical Care Medicine, Department of Medicine, University of Pittsburgh, Pittsburgh, Pennsylvania; 2 Department of Bioengineering, University of Pittsburgh, Pittsburgh, Pennsylvania

**Keywords:** Diastolic dysfunction, echocardiography, ejection fraction, glycolysis, leptin, pressure–volume loop

## Abstract

We have previously reported that 4 weeks of intermittent hypoxia (IH) exposure, mimicking the hypoxic stress of obstructive sleep apnea, produces compensatory increases in left ventricular (LV) contractility in lean C57BL/6J mice. In this study we compared the effects of 4 weeks IH to 4 weeks of sustained hypoxia (SH) on LV function and cardiac glycolysis in lean C57BL/6J mice and obese ob/ob mice at 10–12 weeks of age. The four exposure conditions were IH (nadir O_2_ [5–6%] at 60 cycles/h during the 12 h light period), SH (24 h inspired O_2_ [10%]), and control groups of intermittent air (IA) or room air. Cardiac function was assessed under isoflurane anesthesia (1–2%) by echocardiography and pressure–volume loop analysis and myocardial glycolytic rates were determined ex vivo using radiolabeled ^3^H‐glucose. Lean mice exposed to IH exhibited increases in contractile parameters which were associated with elevated glycolytic rates (3.4 vs. 5.7 *μ*g/*μ*L·g; *P* < 0.05). Ob/ob mice did not show any improvements in contractility after IH. Moreover, cardiac glycolytic rates and LV systolic and diastolic function did not differ from IA ob/ob controls. Following SH exposure, lean mice exhibited increased contractility and glycolytic rates (3.8 vs. 5.7 *μ*g/*μ*L·g; *P* < 0.05), however, LV lumen dimensions were reduced. In contrast, ob/ob mice exposed to SH show compromised systolic and diastolic function associated with unchanging glycolytic rates. These findings demonstrate that, in a murine model of obesity, an inability to increase glycolysis is associated with an absence of an adaptive cardiac response to IH and marked systolic and diastolic dysfunction in response to SH.

## Abstract (precis)

The impact of obesity on left ventricular (LV) function and cardiac glycolytic responses to chronic 4 week exposure to intermittent hypoxia, mimicking the hypoxic stress of obstructive sleep apnea, or sustained hypoxia, mimicking altitude exposure, is unknown. We show that, in a murine model of obesity, an inability to increase glycolysis is associated with an absence of an adaptive cardiac response to intermittent hypoxia and marked systolic and diastolic dysfunction in response to sustained hypoxia.

## Introduction

Hypoxia can result in a variety of cardiac and vascular responses depending on the pattern, severity, and duration of exposure. Rodent models simulating the intermittent hypoxic stress of sleep apnea have exhibited systemic hypertension (Bao et al. 1997; Kanagy et al. 2001) and impaired left ventricular (LV) cardiac function (Chen et al. 2005, 2008, 2010). In contrast, exposure to sustained hypoxia, simulating altitude, is commonly used as a model to induce pulmonary hypertension and right heart hypertrophy (Nattie et al. 1978; Yu et al. 1999; Campen et al. 2005). However, hypoxic exposure may also produce adaptive responses. For example, short periods of intermittent hypoxia can induce ischemic preconditioning, which protect the heart from subsequent infarction (Cai et al. 2003; Beguin et al. 2005). Moreover, we have recently shown using pressure–volume loop analyses that 4 weeks exposure to intermittent hypoxia in lean healthy mice (Naghshin et al. 2009), as well as in mice with heart failure (Naghshin et al. 2012), can increase LV contractility and improve overall cardiac function. Thus, the first goal of this study was to determine the specificity of adaptive cardiac responses by comparing changes in LV function that occur in lean healthy mice with exposure to 4 weeks of intermittent hypoxia versus 4 weeks of sustained hypoxia.

The adaptive or pathologic responses to hypoxia may be dependent on the presence of comorbid conditions, including obesity. We have previously characterized the genetically obese ob/ob mouse as an animal model of obesity hypoventilation syndrome (OHS; O'Donnell et al. 1999) with associated LV hypertrophy (Barouch et al. 2003). The impaired ventilatory control and arterial blood gas homeostasis in OHS may increase vulnerability to subsequent hypoxic stress and exacerbate LV dysfunction. Thus, the second goal of our study was to assess the impact of extended periods of intermittent hypoxia and sustained hypoxia on LV function in a mouse model of obesity with concomitant OHS.

The function of the LV under hypoxic conditions is in part dependent on the metabolic flexibility of cardiac tissue to increase glycolysis to accommodate an increased demand for anaerobic metabolism. Indeed, the ob/ob mouse has an impaired ability to moderate glycolytic metabolism in cardiac tissue under conditions of elevated fatty acid circulating levels (Mazumder et al. 2004). Consequently, our study also aimed to assess cardiac glycolytic rates in ob/ob mice in response to prolonged periods of hypoxic stress. We hypothesized that obese ob/ob mice exhibit a fixed cardiac glycolytic rate and worse LV function than lean mice in response to 4 weeks exposure of either intermittent hypoxia or sustained hypoxia.

## Methods

### Animals

Male C57BL/6J or B6.V‐Lep ob/J (ob/ob) 10‐week‐old mice purchased from the Jackson Laboratory (Bar Harbor, ME) were housed in customized cages as previously described (Polotsky et al. 2006), and exposed to one of four conditions for 4 weeks: an intermittent hypoxia stimulus; intermittent room air control for intermittent hypoxia; sustained hypoxia stimulus; constant room air control for sustained hypoxia. Briefly, a gas control delivery system regulated the flow of room air, nitrogen, and oxygen into the customized cages housing the mice. For the intermittent hypoxia stimulus, a series of programmable solenoids and flow regulators enabled the manipulation of inspired oxygen from 20.9% to 5.0–6.0% over a 30‐sec period with a rapid reoxygenation to room air levels using a burst of 100% O_2_ in the succeeding 30‐sec period. Hypoxic events occurred at a rate of one event per minute throughout the 12 h light period from 8 am to 8 pm. During the 12 h dark period from 8 pm to 8 am, the animals were maintained in a constant undisturbed room air environment. Control animals (sham exposure) for intermittent hypoxia received the same gas flow exposure as the intermittent hypoxia animals, but using only room air. For the sustained hypoxia stimulus, a gas control delivery system regulated the flow of room air and nitrogen to set the inspired oxygen at 10% throughout the 24 h circadian cycle. A group of mice were kept in room air conditions for 4 weeks as controls for sustained hypoxia. Animals were exposed to 28 consecutive days of intermittent hypoxia, intermittent air, sustained hypoxia, or room air prior to the terminal experiment assessing pressure–volume loop function. Our approach in customizing the cages was to allow the animals to live in their normal environment continuously throughout the protocol. All studies were approved by the Institutional Animal Care and Use Committee at the University of Pittsburgh and complied with the American Physiological Society Guidelines for Animal Studies.

### Echocardiography

Transthoracic echocardiography was conducted under light (~1%) isoflurane inhalation anesthesia with the animals breathing spontaneously. Measurements were performed before and after 27 days of exposure to intermittent hypoxia, intermittent air, sustained hypoxia, or room air and 1 day prior to pressure–volume loop analysis and subsequent sacrifice. Short axis M‐ and B‐mode images of the LV were obtained using a VisualSonics 770 machine with a 25‐MHz linear transducer to determine heart rate (HR), end diastolic dimension (EDD), end systolic dimension (ESD), systolic and diastolic anterior wall thicknesses, systolic and diastolic posterior wall thicknesses, and % fractional shortening (%FS), which was calculated as %FS = 100% × (EDD − ESD)/EDD. Echocardiography data collected prior to exposure are included in the Table A1.

### Left ventricular pressure–volume loop analyses

Mice were anesthetized with 1–2% isoflurane in room air via a facemask and positioned on a heating pad with the temperature set to 37.5°C. The anesthetized animal was placed in a supine position. The left external jugular vein was dissected free and catheterized with PE‐10 tubing. The right carotid artery was dissected and exposed. A Millar Mikro‐Tip^®^ conductance catheter (model PVR‐1045, tip size of 1F; Millar Instruments Inc., Houston, TX) was introduced into the artery and advanced into the LV via the aortic valve (Burkhoff et al. 2005). Once steady‐state hemodynamics were achieved, pressure–volume loops were recorded and processed using an MVPS‐400 system (Millar Instruments Inc.). For all animals, parallel conductance (V_P_) was determined individually using a 10–12 *μ*L bolus of 15% saline given through the venous catheter (Georgakopoulos et al. 1998). The cuvette calibration method (Millar Instruments Inc.) was used to calculate the absolute volume data. Using PVAN 3.6 software (Millar Instruments Inc.), the pressure–volume loop data were processed to compute cardiac parameters as described previously (Georgakopoulos et al. 1998; Burkhoff et al. 2005). At the end of the experiment the animals were sacrificed through heart excision.

### Ex vivo cardiac perfusion

Hearts were excised, cannulated through the aorta and perfused with a modified Krebs Henseleit buffer as described previously (Lopaschuk and Barr 1997). Briefly, the buffer contained 118.5 mmol/L NaCl, 25 mmol/L NaHCO_3_, 4.7 mmol/L KCl, 1.2 mmol/L MgSO_4_, 1.2 mmol/L KH_2_P_4_, 2.5 mmol/L CaCl_2_, 0.5 mmol/L EDTA, 11 mmol/L glucose, 0.4 mmol/L palmitate bound to 3% BSA and 0.2 *μ*Ci/mL 5‐[^3^H]glucose (specific activity 13.5 Ci/mmol). During perfusion the solution was continuously gassed with 95% O_2_:5% CO_2_ (pH 7.4) and hearts were allowed to beat spontaneously and temperature maintained at 37.5°C. Following 10 min of stabilization, steady‐state glycolytic rates were determined by averaging the rate of ^3^H_2_O production from perfusate samples collected for 25 min from the beating heart (1 min collection period at 5‐min intervals) and normalized to dry heart weight (McGaffin et al. 2011). ^3^H_2_O was separated from ^3^H‐glucose using ion exchange chromatography as described previously (Lopaschuk and Barr 1997). Calculations were adjusted for volume of collected perfusate per unit time to determine glycolytic rate.

### Statistics

Statistical differences in mean body weight and heart weight between groups were determined using one‐way analysis of variance (ANOVA) and corrected for multiple comparisons with the Bonferroni correction. Strain differences in baseline and final echocardiography, PV loop, and glycolysis data were determined using two‐way ANOVA, constrained to intermittent air and room air control groups. Additionally, two‐way ANOVA was used to determine independent statistical differences (hypoxic exposure main effects) and interactions between exposure conditions (intermittent air and intermittent hypoxia) or (room air and sustained hypoxia) and strain (C57BL/6J vs. ob/ob) for baseline and final echocardiography data, PV loop data, and glycolysis data. Differences between exposure means (intermittent hypoxia vs. intermittent air or sustained hypoxia vs. room air) were determined within strain for all parameters using independent Student's *t*‐test. Values are mean ± SE.

## Results

### Body and heart weights

Body weight and dry heart weight data after 4 weeks exposure to intermittent air, intermittent hypoxia, room air, and sustained hypoxia are shown in Table 1. As expected, lean mice exposed to intermittent hypoxia did not exhibit comparable weight gain throughout the 4 week exposure period as observed in the intermittent air control group (*P* < 0.05), whereas mice in the sustained hypoxia lean group lost approximately 9% body weight compared with the room air lean group (*P* < 0.01). Absolute dry heart weights were lower in both C57BL/6J mice after intermittent hypoxia (*P* < 0.01) and sustained hypoxia (*P* < 0.01), however heart weights adjusted for body weight did not differ from intermittent air and room air controls, respectively. Ob/ob mice exhibited significant obesity at the beginning of exposure, weighing twice as much as age‐matched lean C57BL/6J mice. Unlike lean mice, ob/ob mice exposed to intermittent hypoxia exhibited weight gain comparable to their respective intermittent air controls. Following sustained hypoxia, obese mice did not show any weight gain, and no changes were observed in absolute dry heart weight for either intermittent air or sustained hypoxia ob/ob groups when compared to controls. Finally, there was a trend for body weight adjusted heart weight to be higher in obese mice following sustained hypoxia compared to room air controls (*P* = 0.060).

**Table 1.** Body weight and heart weight in C57BL/6J and ob/ob mice after 4 weeks of intermittent air (IA), intermittent hypoxia (IH), room air (RA), or sustained hypoxia (SH) exposure

Exposure condition	IA	IH	RA	SH
*Lean mice*	*n* = 11	*n* = 11	*n* = 11	*n* = 10
Weight (prior to exposure)	26.8 ± 0.5	26.8 ± 0.6	26.9 ± 0.5	27.7 ± 0.4
Weight (after exposure)	28.6 ± 0.6^†^	26.2 ± 0.4^*^	29.4 ± 0.6^†^	25.2 ± 0.6^†^,^*^
Dry heart weight	0.033 ± 0.001	0.030 ± 0.001^**^	0.034 ± 0.001	0.030 ± 0.001^**^
HW/BW × 1000	1.17 ± 0.03	1.13 ± 0.02	1.14 ± 0.03	1.18 ± 0.02
*Obese mice*	*n* = 10	*n* = 10	*n* = 10	*n* = 9
Weight (prior to exposure)	52.5 ± 1.2	54.5 ± 0.7	52.8 ± 0.8	53.0 ± 0.5
Weight (after exposure)	59.3 ± 1.6^†^	59.2 ± 0.4^†^	60.1 ± 1.0^†^	54.0 ± 1.3^**^
Dry heart weight	0.037 ± 0.006	0.038 ± 0.006	0.037 ± 0.004	0.037 ± 0.004
HW/BW × 1000	0.65 ± 0.04	0.65 ± 0.03	0.62 ± 0.02	0.69 ± 0.02

Values are mean ± SE.

*P < 0.05, **P < 0.01 between IA and IH groups or RA and SH groups within strain, ^†^*P* < 0.05 between body weight (prior to exposure) and body weight (after exposure) by one‐way ANOVA with the Bonferroni correction.

### Altered LV function and cardiac glycolysis in leptin‐deficient, obese mice at baseline

**Figure 1.**
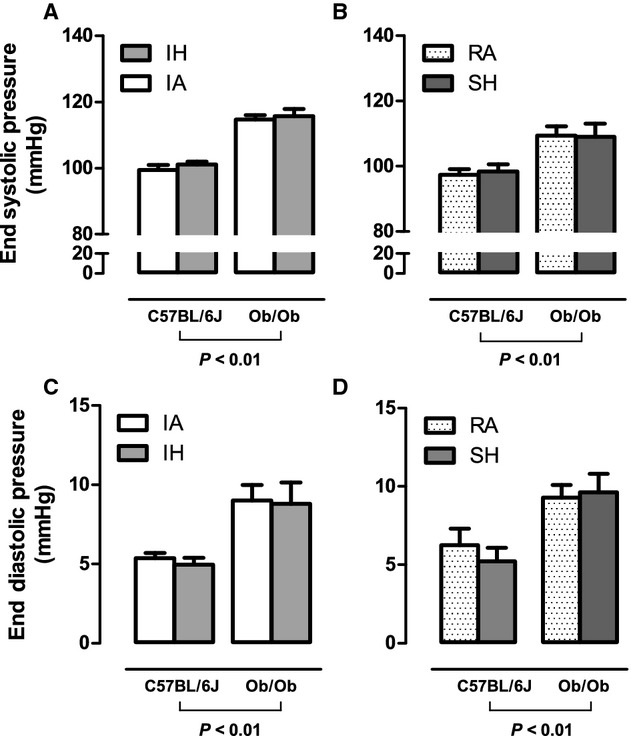
The effects of 4 weeks exposure to intermittent air (IA), intermittent hypoxia (IH), room air (RA), and sustained hypoxia (SH) on LV developed pressures in lean and leptin‐deficient obese mice. Mean ± SE values for: end systolic pressure (ESP; A and B) and end diastolic pressure (EDP; C and D) determined by steady‐state pressure–volume loops in either C57BL/6J or ob/ob mice after 4 weeks of IA, IH, RA, or SH. Significant strain effects were determined by two‐way ANOVA and are reported on the figure. No hypoxia exposure effects and no significant interactions were found.

**Figure 2.**
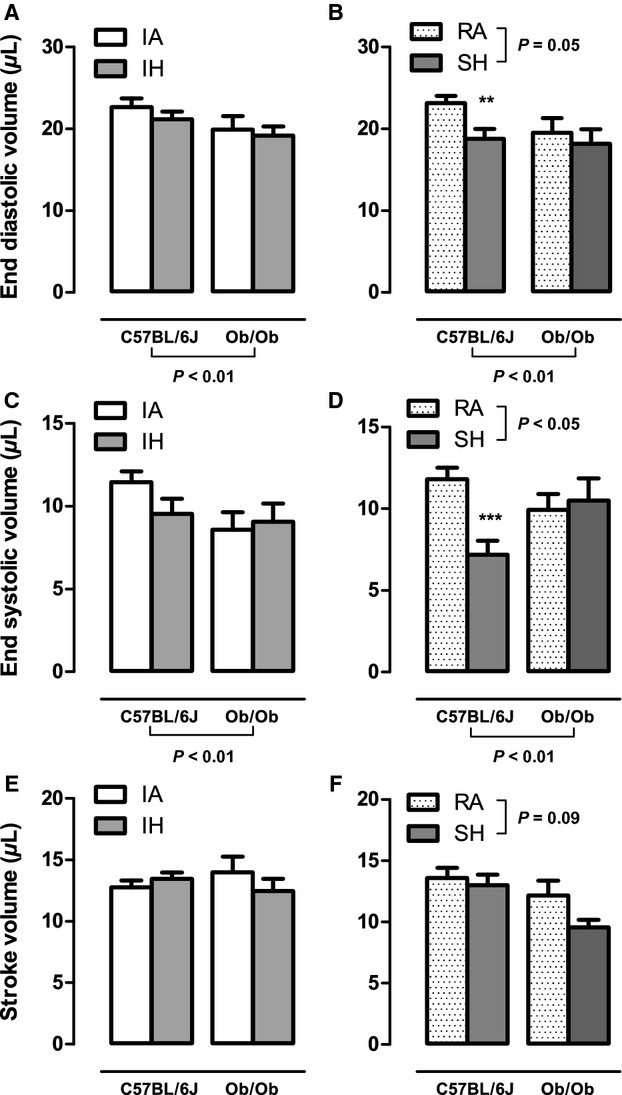
The effects of 4 weeks exposure to intermittent air (IA), intermittent hypoxia (IH), room air (RA), and sustained hypoxia (SH) on left ventricular volumes in lean and leptin‐deficient obese mice. Mean ± SE values for: end diastolic volume (EDV; A and B), end systolic volume (ESV; C and D), and stroke volume (SV; E and F) determined by steady‐state pressure–volume loops in either C57BL/6J or ob/ob mice after 4 weeks of IA, IH, RA, and SH. Significant strain and hypoxia exposure effects were determined by two‐way ANOVA and are reported on the figure. Two‐way ANOVA showed a significant interaction effect between mouse strain and SH hypoxia exposure for ESV (*P* < 0.05). ***Significant difference (*P* < 0.001). **Significant difference (*P* < 0.01). *Significant difference (*P* < 0.05).

Baseline and postexposure echocardiography data for all groups are included in Table A1 (Appendix) and Table 2, respectively. Baseline echocardiography data showed that ob/ob mice exhibited a trend for FS to be elevated (*P* = 0.066) and larger systolic posterior and anterior wall thicknesses when compared to lean C57BL/6J mice (Table A1). Using pressure–volume analysis we determined that ob/ob mouse control groups (intermittent air and room air) exhibited a hypercontractile state defined by significantly elevated levels of end systolic pressure (Fig. 1A and B), preload adjusted maximal power (Fig. 4E and F), and dP/dt_max_ (Fig. 4A and B) together with elevated end diastolic pressure (Fig. 1C and D) and dP/dt_min_ (Fig. 5C and D) when compared to lean C57BL/6J intermittent air and room air controls. This increase in systolic and diastolic pressure and power‐based parameters is consistent with our echocardiography data and was accompanied by slightly lower but statistically significant end diastolic volume (Fig. 2C and D) and end systolic volume (ESV; Fig. 2C and D) in the presence of maintained stroke volume (Fig. 2E and F). Baseline cardiac glycolytic rates measured for ob/ob mice exposed to room air and intermittent air were both higher than cardiac glycolytic rates measured for lean C57BL/B6 mice exposed to the same conditions (Fig. 6).

**Table 2.** Echocardiographic assessment of LV function in C57BL/6J and ob/ob mice after 4 weeks of intermittent air (IA), intermittent hypoxia (IH), room air (RA), or sustained hypoxia (SH) exposure

Baseline	C57BL/6J		ob/ob		Hypoxia effect	Interaction	Strain effect
	IA	IH	IA	IH			
HR, beats/min	545 ± 22	559 ± 11	506 ± 25	489 ± 14			
FS, %	27.1 ± 2.1	31.8 ± 1.5	32.7 ± 3.0	33.9 ± 1.4			*P* < 0.05
EDD, mm	3.87 ± 0.16	3.78 ± 0.10	4.04 ± 0.11	3.94 ± 0.09			
ESD, mm	2.85 ± 0.18	2.59 ± 0.12	2.74 ± 0.18	2.61 ± 0.11			
PWTd, mm	0.80 ± 0.04	0.75 ± 0.03	0.81 ± 0.04	0.84 ± 0.04			
PWTs, mm	1.08 ± 0.06	1.05 ± 0.06	1.15 ± 0.04	1.18 ± 0.04			*P* < 0.05
SWTd, mm	0.91 ± 0.04	0.85 ± 0.02	1.00 ± 0.08	0.91 ± 0.04			
SWTs, mm	1.22 ± 0.05	1.22 ± 0.03	1.40 ± 0.09	1.34 ± 0.08			*P* < 0.01

Baseline	C57BL/6J		ob/ob		Hypoxia effect	Interaction
	RA	SH	RA	SH		
HR, beats/min	515 ± 16	570 ± 13	493 ± 26	458 ± 25		*P* < 0.05
FS, %	29.2 ± 1.4	34.0 ± 1.8	33.3 ± 1.2	32.3 ± 2.8		
EDD, mm	3.93 ± 0.01	3.51 ± 0.09	4.08 ± 0.08	3.72 ± 0.22	*P* < 0.01	
ESD, mm	2.78 ± 0.01	2.27 ± 0.12	2.73 ± 0.09	2.43 ± 0.19	*P* < 0.005	
PWTd, mm	0.79 ± 0.03	0.86 ± 0.06	0.84 ± 0.03	0.83 ± 0.04		
PWTs, mm	1.07 ± 0.03	1.25 ± 0.08	1.14 ± 0.04	1.15 ± 0.05		
SWTd, mm	0.84 ± 0.04	0.87 ± 0.03	1.03 ± 0.04	0.93 ± 0.09		
SWTs, mm	1.16 ± 0.03	1.24 ± 0.04	1.44 ± 0.05	1.33 ± 0.11		

Values are means ± SE.

HR, heart rate; FS, fractional shortening; EDD, end diastolic dimension; ESD, end systolic dimension; PWTd, posterior wall thickness diastole; PWTs, posterior wall thickness systole; SWTd, septal wall thickness diastole; SWTs, septal wall thickness systole. Exposure effect, interaction and strain effect were determined by two‐way ANOVA.

### Lean C57BL/6J mice, but not obese ob/ob mice, exhibit enhanced contractility and increased cardiac glycolytic rates in response to 4 weeks of intermittent hypoxia

**Figure 3.**
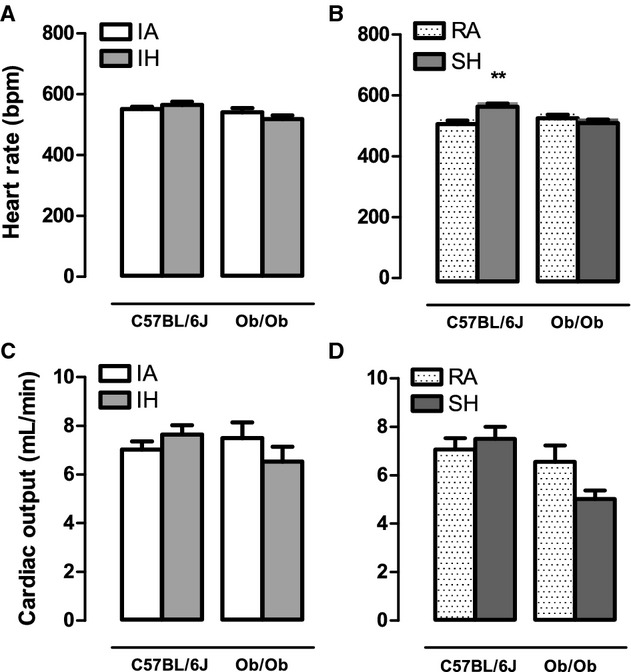
The effects of 4 weeks exposure to intermittent air (IA), intermittent hypoxia (IH), room air (RA), and sustained hypoxia (SH) on overall cardiac function in lean and leptin‐deficient obese mice. Mean ± SE values for: heart rate (HR; A and B), and cardiac output (CO; C and D) determined by steady‐state pressure–volume loops in either C57BL/6J or ob/ob mice after 4 weeks of IA, IH, RA, and SH. No significant strain or hypoxia exposure effects were determined by two‐way ANOVA. Two‐way ANOVA showed a significant interaction effect between mouse strain and SH hypoxia exposure for HR (*P* < 0.01). **Significant difference (*P* < 0.01).

**Figure 4.**
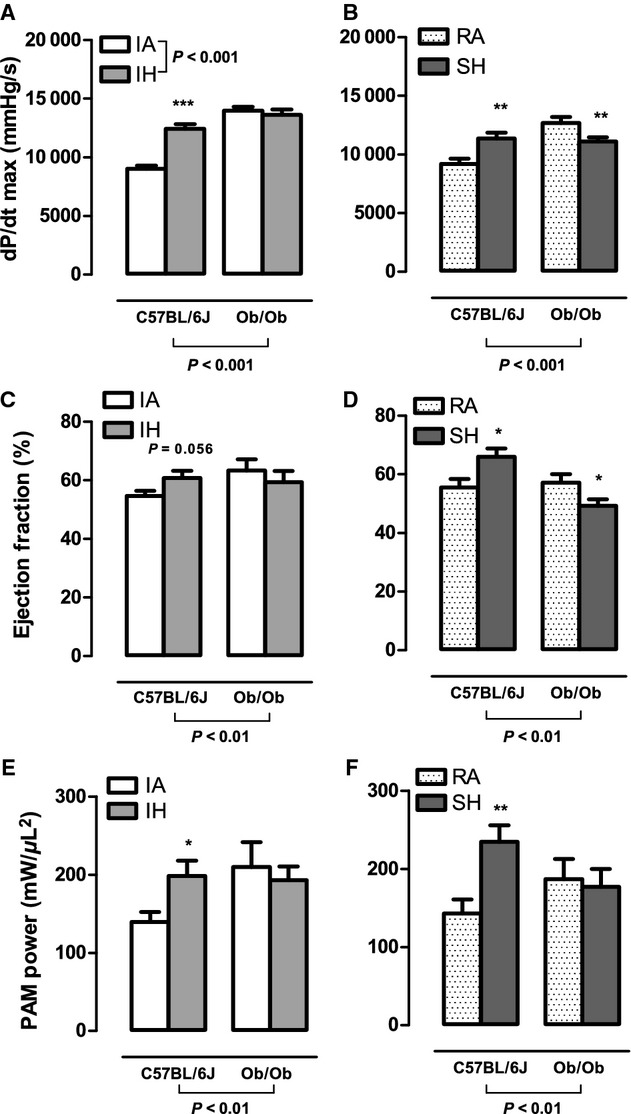
The effects of 4 weeks exposure to intermittent air (IA), intermittent hypoxia (IH), room air (RA), and sustained hypoxia (SH) on systolic function in lean and leptin‐deficient obese mice. Values are mean ± SE for: maximum rate of pressure change (dP/dt_max_; A and B), ejection fraction (EF; C and D) and preload adjusted maximal power (PAMP; E and F) determined by steady‐state pressure–volume loops in either C57BL/6J or ob/ob mice after 4 weeks of IA, IH, RA, or SH. Significant strain and hypoxia exposure effects were determined by two‐way ANOVA and are reported on the figure. Two‐way ANOVA showed significant interaction effects between strain and SH exposure for dP/dt_max_ (*P* < 0.001), PAMP (*P* < 0.05), and EF (*P* < 0.01). Two‐way ANOVA also showed a significant interaction between strain and IH exposure for dP/dt_max_ (*P* < 0.001). *Significant difference (*P* < 0.05). **Significant difference (*P* < 0.01).

**Figure 5.**
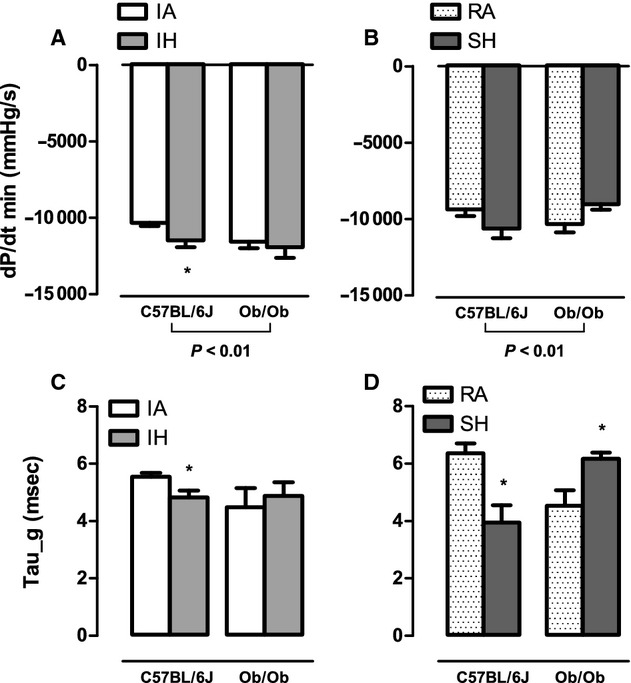
The effects of 4 weeks exposure to intermittent air (IA), intermittent hypoxia (IH), room air (RA), and sustained hypoxia (SH) on diastolic function in lean and leptin‐deficient obese mice. Values are mean ± SE for: minimum rate of pressure change (dP/dt_min_; A and B) and time constant *Tau* (*Tau*; C and D) determined by steady‐state pressure–volume loops in either C57BL/6J or ob/ob mice after 4 weeks of IA, IH, RA, or SH. Significant strain effects were determined by two‐way ANOVA and are reported on the figure. No hypoxia exposure effects were determined. Two‐way ANOVA showed significant interaction effects between strain and SH exposure for both Tau (*P* < 0.001) and dP/dt_min_ (*P* < 0.05). *Significant difference (*P* < 0.05). **Significant difference (*P* < 0.01).

**Figure 6.**
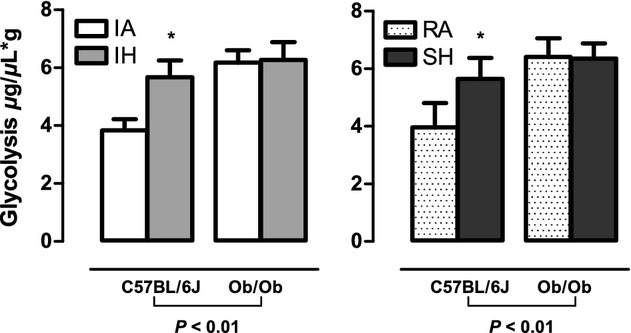
The effects of 4 weeks exposure to intermittent air (IA), intermittent hypoxia (IH), room air (RA), and sustained hypoxia (SH) on glycolytic cardiac rates in lean and leptin‐deficient obese mice. Values are mean ± SE for cardiac glycolytic rate determined by ex vivo cardiac perfusion in either C57BL/6J or ob/ob mouse hearts after 4 weeks of IA, IH, RA, or SH. Significant strain effects were determined by two‐way ANOVA and are reported on the figure. No significant hypoxia exposure effects or interactions were determined. *Significant difference (*P* < 0.05).

In lean mice, global cardiac function was preserved after 4 weeks of intermittent hypoxia exposure when compared to intermittent air (Fig. 3). Indexes of contractility were significantly augmented following 4 weeks of intermittent hypoxia including dP/dt_max_ (Fig. 4A) and preload adjusted Max_Power (Fig. 4E), with a strong trend for an increase in ejection fraction (*P* = 0.056; Fig. 4C). No significant changes were observed in SV and EDV (Fig. 2E and A), but there was a weak trend for a decrease in ESV (IA: 11.45 *μ*L vs. IH: 9.54 *μ*L; *P* = 0.103; Fig. 2C) that likely accounts for the response in ejection fraction. Consistent with the pressure–volume loop data, echocardiography data showed a trend (*P* = 0.066) for an increased FS when compared to intermittent air mice. Parameters characterizing diastolic function also showed improvements following intermittent hypoxia with elevated dP/dt_min_ (Fig. 5A) and a decrease in relaxation time constant Tau (Fig. 5C). The enhanced LV contractile response to intermittent hypoxia was accompanied by significantly elevated cardiac glycolytic rates (Fig. 6) indicating a greater capacity for anaerobic metabolism in the intermittent hypoxia‐exposed hearts.

In ob/ob mice, pressure–volume loop analyses did not reveal any significant intermittent hypoxia‐induced changes in parameters reflecting global cardiac function, contractility, or diastolic function compared to ob/ob intermittent air controls (Figs 1–5). Similarly, echocardiography did not show any changes in heart structure or function after 4 weeks of intermittent hypoxia exposure (Table 2). Finally, glycolytic rates were unchanged between ob/ob mice exposed to intermittent hypoxia and ob/ob mice exposed to intermittent air (Fig. 6).

### Obese, but not lean mice, develop LV systolic and diastolic dysfunction in response to sustained hypoxia

In lean mice, echocardiography demonstrated that LV systolic and diastolic lumen dimensions (Table 2) are reduced in response to 4 weeks of sustained hypoxia. Correspondingly, pressure–volume loop analysis showed lower end diastolic volume (Fig. 2B) and ESV (Fig. 2D) in sustained hypoxia mice compared to room air controls. Despite lower working LV volumes, overall cardiac function as defined by stroke volume (Fig. 2F) and cardiac output (Fig. 3D) was comparable between sustained hypoxia and room air mice. Ejection fraction (Fig. 4D), dP/dt_max_ (Fig. 4B), and PAMP (Fig. 4F) were all significantly elevated in lean mice following sustained hypoxia exposure, indicating increased contractility. However, heart rate in sustained hypoxia mice was significantly higher than room air controls (Fig. 3B), likely contributing to the measured rise in dP/dt_max_. Thus, the heightened contractility observed in the sustained hypoxia mouse group is in part attributed to a larger HR, a pattern of response that was not seen with the increases in contractility seen in the lean mice exposed to intermittent hypoxia (Fig. 4A, C, and E). Diastolic function was also improved following sustained hypoxia, as evident by an increase in dP/dt_min_ (Fig. 5B) and a decrease in the relaxation time constant Tau (Fig. 5D). As indicated for contractile function, the increased heart rate likely contributed to the observed changes in improved diastolic function. Finally, in a similar manner to what we observed in intermittent hypoxia, lean mice exposed to sustained hypoxia show an increased capacity for anaerobic metabolism with glycolytic rates higher than in room air controls (Fig. 6).

In ob/ob mice there was a reduction in both end diastolic and ESDs with exposure to sustained hypoxia (Table 2), a pattern of response quantitatively similar to the effect of sustained hypoxia on LV dimensions in lean mice (Table 2). However, there were strong trends for stroke volume (*P* = 0.079) and cardiac output (*P* = 0.070) to be reduced in obese mice after sustained hypoxia (Figs. 2F and 3D) indicating impaired overall cardiac function. Decreases in dP/dt_max_ (Fig. 4B) and ejection fraction (Fig. 4F) in sustained hypoxia compared to room air animals show a significant deterioration in contractility. Furthermore, a decrease in dP/dt_min_ and an increase in Tau in ob/ob mice demonstrate the development of significant diastolic dysfunction in response to sustained hypoxia (Fig. 5B and D). Similar to the obese intermittent hypoxia group, glycolytic metabolism remained unchanged following 4 weeks of sustained hypoxia in the ob/ob mice (Fig. 6).

## Discussion

The cardiac responses to hypoxia are complex and necessarily dependent on exposure patterns and duration. Contrary to expectations derived from clinical studies in OSA patients (Shahar et al. 2001; Ancoli‐Israel et al. 2003), and some published data in rats and mice (Chen et al. 2005, 2008, 2010), we have previously reported that 4 weeks exposure to intermittent hypoxia in mice does not lead to impaired cardiac function (Naghshin et al. 2009, 2012). In fact, after 4 weeks of intermittent hypoxia we see an apparent adaptive increase in LV contractility in lean mice (Naghshin et al. 2009, 2012). In this study, one goal was to determine the specificity of this LV response to intermittent hypoxia in lean healthy mice. We now show that 4 weeks of sustained hypoxia leads to similar increases in contractility in lean mice, albeit in the presence of reduced systolic and diastolic LV volumes. We go on to show an enhanced glycolytic capacity in both intermittent hypoxia and sustained hypoxia exposed hearts, providing the capability of enhanced anaerobic metabolism in response to both forms of hypoxic stress. A second goal of our study was to determine the impact of intermittent hypoxia and sustained hypoxia on LV function in a rodent model with underlying blood gas disturbances due to OHS. Interestingly, we found that obese mice exposed to intermittent hypoxia did not exhibit an adaptive response similar to the lean mice, but rather maintained a baseline phenotype that was associated with an inability to increase cardiac glycolysis. Furthermore, sustained hypoxia markedly disrupted LV function with decrements in global cardiac function, as well as systolic and diastolic function, again associated with unchanged glycolytic capacity. In the discussion that follows, we discuss the implications of our physiologic findings and their potential relevance in the clinical setting.

### Adaptive cardiac responses to hypoxia in lean mice

The LV cardiac responses in lean mice exposed to 4 weeks of intermittent hypoxia reported in this study are in agreement with previous data from our laboratory showing that 4 weeks of intermittent hypoxia leads to an adaptive increase in contractility without hypertrophy in C57BL/6J mice (Naghshin et al. 2009), FVB mice (Naghshin et al. 2012), and even in mice with a natural history of heart failure (Naghshin et al. 2012). We now show that the increase in contractility is linked, potentially mechanistically, to increased cardiac glycolytic rates. Further, this study extends the investigation to determine cardiac responses to 4 weeks of sustained hypoxia. We show similar LV contractile responses to sustained hypoxia as were seen in intermittent hypoxia exposure, including an ability to increase glycolysis. However, differences in working LV volumes, cardiac lumen diameters, and heart rates were observed between sustained hypoxia and intermittent hypoxia. Potentially, changes in preload (Reeves et al. 1987) and afterload, intraventricular septal deviation (Tanaka et al. 1980; Louie et al. 1992), or diuresis (Bartsch and Gibbs 2007) could account for the decrease in LV volumes after sustained hypoxia exposure. Potentially, a greater ability of lean mice to increase breathing frequency to chemoreceptor stimulation could lead to increased dehydration and subsequent reduced plasma volumes during sustained hypoxia compared to obese mice (O'Donnell et al. 1999; Polotsky et al. 2001). A lack of change in end systolic pressure indicates that increased afterload is an unlikely contributing factor. We found no evidence of pericardial constraints in echocardiography analyses, potentially excluding reduced preload as the cause of reduced LV volumes. However, we did not measure diuresis or plasma volume, which have previously been shown to be diminished over the first few weeks of sustained hypoxia associated with high‐altitude exposure (Pugh 1964; Fusch et al. 1996), and therefore likely play a role in the reduction in lumen volumes.

### Ob/ob mice exhibit differential cardiac responses to intermittent hypoxia and sustained hypoxia

In response to intermittent hypoxia, the ob/ob mouse model of OHS did not exhibit an increase in LV contractility, whereas sustained hypoxia caused a marked decrease in contractility. Exposure to 3 or more weeks of sustained hypoxia in mice is commonly used as a model of experimental right heart hypertrophy and pulmonary hypertension due to the development of increased pulmonary vascular resistance (Nattie et al. 1978; Zhong et al. 2002; Campen et al. 2005). In ob/ob mice, both right heart hypertrophy and pulmonary hypertension are exacerbated by sustained hypoxia relative to responses in lean mice (Irwin et al. 2007). Although we did not assess right heart function, we cannot discount that right ventricular hypertrophy accompanied by pulmonary hypertension contributed to the LV systolic dysfunction that develops in ob/ob mice in response to sustained hypoxia.

We have previously shown that disruption of cardiac‐specific leptin signaling, but not obesity per se impairs the ability of mice to increase glycolysis in response to a cardiac ischemic hypoxic stress (McGaffin et al. 2011). An inability to increase glycolysis could disrupt the balance between oxygen demand and cardiac work, potentially compromising cardiac performance. Additionally, under baseline conditions, ob/ob mice have elevated LV pressures and heightened contractility relative to lean mice, which taken together with an inability to increase glycolysis could account for the absence of an adaptive contractile response to intermittent hypoxia in the obese mice. Mechanistically, carbohydrate substrate utilization in the heart has previously been proposed as a link between contractile efficiency and cardiac workloads during hypoxic exposure (Hutter et al. 1984; Burkhoff et al. 1991; Korvald et al. 2000). Indeed, glycolytic blockade has been shown to exacerbate the decrements in contractility observed in ischemia–reperfusion studies (Apstein et al. 1978; Palmer et al. 2004). Moreover, glycolysis‐mediated buffering of phosphate metabolites can enhance contractile efficiency during high‐workload conditions (Harrison et al. 2003). Thus, the inability of the ob/ob mice to mimic the increase in LV contractility evident in lean mice may in part be related to an inability to increase cardiac glycolysis.

Glycolysis can also play an important role in cardiac relaxation mechanics. Compartmentalized glycolytic flux and the enzymes involved in the production of glycolytic‐derived ATP have been shown to be closely linked to trans‐sarcolemal ion transport (Pierce and Philipson 1985) through the Na‐K ATPase (Weiss and Hiltbrand 1985; Campbell and Paul 1992) and ATP‐sensitive K+ channels (Weiss and Lamp 1987). Additionally, glycolytic‐derived ATP has been associated with restoring cytoplasmic calcium homeostasis in the postischemic myocardium (Jeremy et al. 1992). Taken together with our data, we propose that a glycolytic ceiling effect may potentially contribute to the LV diastolic dysfunction that develops in ob/ob mice when faced with chronic sustained hypoxic stress, although the absence of a similar LV dysfunction with intermittent hypoxia exposure would suggest a unique interaction between limited glycolysis and the specific regimen of hypoxia.

### Study limitations

We did not perform assessment of cAMP activity or protein expression in cardiac tissue after exposure to hypoxia, limiting our ability to associate cellular responses with changes in cardiac function or glycolysis. However, the OHS mouse model of obesity is caused by a global leptin deficiency that removes inhibition on hypothalamic centers controlling satiety. In addition to satiety control (Friedman and Halaas 1998; Elmquist et al. 1999), leptin signaling has been shown to activate adrenergic signaling (Haynes et al. 1997; Satoh et al. 1999; Minhas et al. 2005) to the heart. Given that our previous studies have shown that the adaptive response to intermittent hypoxia is partially mediated through *β*‐adrenergic signaling (Naghshin et al. 2009), we speculate that the adaptive response to intermittent hypoxia we see in lean, but not obese, mice is dependent on intact leptin signaling pathways stimulating adrenergic pathways. Moreover, we are unable with the ob/ob model to separate obesity from leptin deficiency as an individual single factor contributing to the divergent effects of the hypoxia exposures on LV function between the lean and obese mice. Our decision to study the ob/ob model of OHS rather than C57BL/6J mice with diet‐induced obesity, which exhibit normal ventilatory control, was to maximize the clinical relevance of our study since the presence of underlying blood gas disturbances is a common comorbidity in hypoxic disorders such as OSA and chronic obstructive pulmonary disease.

### Clinical implications

Studies investigating the effects of high‐altitude exposure on the cardiovascular system have had a major focus on right heart dynamics (Ostadal and Kolar 2007) and these investigations have provided the basis for clinical recommendations for individuals assenting to altitude (Bartsch and Gibbs 2007; Rimoldi et al. 2010). Our study highlights the possibility of pathophysiologic effects on LV dynamics resulting from the hypoxic stress of high‐altitude exposure and obesity, suggesting that obesity could represent a cardiac risk factor or contraindication for long‐term altitude exposure. Although not studied in the clinical setting, any possible mechanical or metabolic advantage the heart develops in response to the intermittent hypoxia component of OSA may be attenuated by the presence of obesity or leptin insensitivity. Moreover, as previously proposed, the cardiac adaptations to 4 weeks exposure of intermittent hypoxia are consistent with the subsequent development of ventricular hypertrophy, and it is predicted that a more chronic exposure, modeling the progressive nature of clinical OSA, will ultimately lead to a failing LV.

## Acknowledgments

The authors would like to thank Kenneth McGaffin for guidance with the metabolic analysis procedure and pressure–volume loop surgical technique, William G. Witham for assistance with the metabolic analysis procedure, and finally, Lia C. Romano for assisting with echocardiography measurements.

## Conflict of Interest

None declared.

## Appendix

**A1:** Echocardiographic assessment of LV function in C57BL/6J and ob/ob mice at baseline prior to intermittent air, intermittent hypoxia, room air, or sustained hypoxia exposure

Baseline	C57BL/6J		ob/ob		Hypoxia effect	Interaction effect	Strain
	IA	IH	IA	IH			
HR, beats/min	514 ± 15	517 ± 10	497 ± 18	478 ± 13			
FS, %	25.6 ± 2.4	25.4 ± 1.5	31.5 ± 1.9	33.7 ± 2.7			*P* < 0.05
EDD, mm	3.98 ± 0.09	4.05 ± 0.10	4.10 ± 0.07	4.00 ± 0.11			
ESD, mm	2.97 ± 0.14	3.03 ± 0.12	2.82 ± 0.12	2.67 ± 0.16			
PWTd, mm	0.82 ± 0.05	0.81 ± 0.03	0.81 ± 0.04	0.75 ± 0.02			
PWTs, mm	1.08 ± 0.07	1.07 ± 0.06	1.11 ± 0.07	1.07 ± 0.05			*P* < 0.05
SWTd, mm	0.89 ± 0.04	0.86 ± 0.04	1.01 ± 0.03	0.96 ± 0.04			
SWTs, mm	1.19 ± 0.07	1.15 ± 0.04	1.45 ± 0.04	1.34 ± 0.05			*P* < 0.01

Baseline	C57BL/6J		ob/ob		Hypoxia effect	Interaction
	RA	SH	RA	SH		
HR, beats/min	505 ± 17	519 ± 13	468 ± 14	443 ± 15		
FS, %	27.1 ± 1.3	28.5 ± 1.6	31.9 ± 3.0	31.1 ± 0.7		
EDD, mm	3.95 ± 0.06	3.95 ± 0.11	4.03 ± 0.12	4.02 ± 0.12		
ESD, mm	2.88 ± 0.09	2.83 ± 0.11	2.77 ± 0.18	2.77 ± 0.10		
PWTd, mm	0.75 ± 0.02	0.78 ± 0.03	0.74 ± 0.03	0.81 ± 0.05		
PWTs, mm	0.99 ± 0.02	1.03 ± 0.03	1.03 ± 0.07	1.10 ± 0.04		
SWTd, mm	0.87 ± 0.05	0.90 ± 0.04	0.99 ± 0.04	0.94 ± 0.06		
SWTs, mm	1.19 ± 0.06	1.21 ± 0.02	1.37 ± 0.07	1.29 ± 0.07		

Values are means ± SE. HR, heart rate; FS, fractional shortening; EDD, end diastolic dimension; ESD, end systolic dimension; PWTd, posterior wall thickness diastole; PWTs, posterior wall thickness systole; SWTd, septal wall thickness diastole; and SWTs, septal wall thickness systole. Exposure effect, interaction and strain effect were determined by two‐way ANOVA.
